# Natural Killer Cells and Regulatory T Cells Cross Talk in Hepatocellular Carcinoma: Exploring Therapeutic Options for the Next Decade

**DOI:** 10.3389/fimmu.2021.643310

**Published:** 2021-04-30

**Authors:** Amber G. Bozward, Frazer Warricker, Ye H. Oo, Salim I. Khakoo

**Affiliations:** ^1^ Centre for Liver and Gastroenterology Research and National Institute for Health Research Biomedical Research Centre (NIHR BRC) Birmingham, Institute of Immunology and Immunotherapy, University of Birmingham, Birmingham, United Kingdom; ^2^ Centre for Rare Diseases, European Reference Network Centre- Rare Liver, Birmingham, United Kingdom; ^3^ The School of Clinical and Experimental Sciences, Faculty of Medicine, University of Southampton, Southampton, United Kingdom; ^4^ NIHR Biomedical Research Centre, The School of Clinical and Experimental Sciences, University of Southampton, Southampton, United Kingdom; ^5^ Liver Transplant and Hepatobiliary Unit, University Hospital of Birmingham NHS Foundation Trust, Birmingham, United Kingdom

**Keywords:** liver, NK cells, regulatory T cells, hepatocellular carcinoma, tumour microenvironment, GMP cell therapy

## Abstract

Despite major advances in immunotherapy, hepatocellular carcinoma (HCC) remains a challenging target. Natural Killer (NK) cells are crucial components of the anti-HCC immune response, which can be manipulated for immunotherapeutic benefit as primary targets, modulators of the tumour microenvironment and in synchronising with tumour antigen specific effector CD8 cells for tumour clearance. Regulatory T cells shape the anti-tumour response from effector T cells *via* multiple suppressive mechanisms. Future research is needed to address the development of novel NK cell-targeted immunotherapy and on restraining Treg frequency and function in HCC. We have now entered a new era of anti-cancer treatment using checkpoint inhibitor (CPI)-based strategies. Combining GMP-NK cell immunotherapy to enhance the frequency of NK cells with CPI targeting both NK and CD8 T cells to release co-inhibitory receptors and enhance the cells anti-tumour immunity of HCC would be an attractive therapeutic option in the treatment of HCC. These therapeutic approaches should now be complemented by the application of genomic, proteomic and metabolomic approaches to understanding the microenvironment of HCC which, together with deep immune profiling of peripheral blood and HCC tissue before and during treatment, will provide the much-needed personalised medicine approach required to improve clinical outcomes for patients with HCC.

## The Liver as an Organ of Immunotolerance

The liver is a unique lymphoid organ which plays a key role in the immunological function of the human body. Embryologically, the human liver is derived from the endoderm layer and resides between two venous circulatory systems; the portal vein, receiving venous flow from the gastrointestinal tract and the systemic venous circulation. The liver has a unique immunological environment containing both professional antigen presenting cells, dendritic cells and Kupffer cells (resident hepatic macrophages) as well as non-professional antigen presenting cells (sinusoidal endothelial cells and biliary epithelium) (1). There is also an abundance of natural killer (NK) cells, innate lymphoid cells (ILCs) and innate mucosa associate invariant T (MAIT) cells, all of which play integral roles in the innate immune response of the liver.

The liver is constantly filtering harmful and harmless antigens from the gut acting as an immunological firewall (1). Hepatic tolerance to gut antigens is achieved by a combination of both immune cells and parenchymal cells. The constant exposure to gut-derived bacteria triggers a downregulation of Toll-like receptor 4 (TLR4) on the hepatic sinusoidal endothelial cells (HSEC). Liver-resident dendritic cells (DC) have distinct properties that promote tolerance rather than an immune response. These tolerogenic DCs secrete anti-inflammatory cytokines such as interleukin 10 (IL-10) and transforming growth factor β (TGF-β) to dampen the immune response and can promote T-cell “hyporesponsiveness” (2, 3). Kupffer cells (KCs), known as sinusoidal firewalls, also contribute to hepatic tolerance by continuously phagocytosing the microbial products from the portal vein (4). Hepatic regulatory T cells (Treg) also play a crucial role in maintaining the tolerogenic environment by continuously controlling the cytokine production and proliferation of intrahepatic auto-reactive effector CD4 and CD8 T cells (5).

The liver is known as a “graveyard” of immune cells due to apoptosis of activated lymphocyte populations (6). It can mount an effective immune response to invading pathogens and cancer cells or when there is insult or loss of peripheral self-tolerance in immune-mediated liver injury. The balance between immunity and tolerance is established by competition for primary activation of effector T cells between the liver and its draining lymphoid tissues. For example, naive CD8^+^ T cells, activated within liver-draining portal lymph nodes are capable of mediating hepatitis, while cells undergoing primary activation within the liver exhibit defective cytotoxic function and do not induce hepatocellular injury (7). The hepatic immune response will depend on the nature of the injury. In acute hepatic injury due to, for example, viral infection or drug-induced liver injury, an innate immune cells infiltrate appears important, with eosinophil or neutrophils and natural killer (NK) cells being the predominant immune cells. Adaptive immune cells are dominant in chronic injury resulting from, for example, chronic hepatitis B and C infection, alcoholic and non-alcoholic steatohepatitis, or autoimmune hepatitis. In the context of HCC, both innate cells such as NK cells and adaptive T cells are involved in auto-tumour immunity. In general, the balance of effector and regulatory T cells determines the outcome of inflammation, either resolution or chronic active hepatitis (8).

## Hepatocellular Carcinoma

In 2018, HCC was the sixth most common neoplasm diagnosed globally and was the fourth leading cause of cancer related death. In the vast majority of cases, primary hepatocellular carcinoma (HCC) arises on a background of cirrhosis, driven by chronic inflammation from a number of causes. These include viral (hepatitis B and hepatitis C) and non-viral (non-alcoholic fatty liver disease and alcoholic liver disease). Improved treatments for chronic viral hepatitis, coupled with the global epidemic of obesity imply that in the coming years the global epidemiology of HCC may shift from infectious to non-infectious causes.

The recent observation that hepatocytes within cirrhotic nodules have a higher mutational load than normal liver characterizes cirrhosis as a truly pre-malignant condition (9). Coexistent with this is the immune dysfunction associated with cirrhosis. This is characterized by evidence of peripheral activation of circulating immunocytes exposed to higher than normal levels of bacterial antigens, but a more profound central tolerance. A healthy liver is a tolerogenic organ, however this state is exacerbated by changes in immune sub-populations and their dysfunction in cirrhosis. The combination of an increased hepatic mutational burden together with decreased immune surveillance underpins the development of HCC.

## Immune Dysfunction in Cirrhosis

As described above, in a healthy liver, immune system homeostasis is achieved through immunosurveillance of its dual blood supply (portal vein and hepatic artery), protecting the host from microbe-associated molecular patterns and damage-associated molecular patterns (MAMPs and DAMPs respectively) from the gut (10). Concurrently the liver displays features of local immune tolerance to non-pathogenic material and helps mediate the appropriate immune response through the synthesis of pro-inflammatory and anti-inflammatory cytokines (11). The immune system thus plays a decisive role in both the pathogenesis of cirrhosis and subsequent immune dysfunction. Chronic factors including infection, alcohol and obesity, inflict persistent hepatocyte damage leading to fibrosis *via* hepatic stellate cell (HSC) activation (12). Disease specific alterations may compound these factors and augment the rate of fibrosis progression. Once cirrhosis becomes established, the liver loses its ability to appropriately protect the body from pathogens as a consequence of disordered immune cell activation termed “cirrhosis-associated immune dysfunction”.

In early cirrhosis, changes in the intrahepatic immune compartment are often due to persistent innate immune cell stimulation. As disease progression ensues, ultimately leading to decompensated cirrhosis, immune hyporesponsiveness and increased tolerance develops (13). This is driven by the innate immune system in which long-term exposure of toll-like receptors to bacterial products such as lipopolysaccharide, can lead to a dampened innate immune response (14). Cirrhosis is associated with a multitude of abnormalities in the innate and adaptive arms of the immune system leading to a generalised immune hyporesponsiveness. The reticulo-endothelial system becomes compromised in the context of cirrhosis as a result of fibrotic damage to the sinusoidal vascular space. This leads to capillarisation, porto-systemic shunts and loss of the KC population, which are also dysfunctional, with impaired phagocytosis (10, 15). Consequently, the capacity for clearance of endotoxin and microbes is attenuated, lowering the threshold for bacterial infection. This dysfunction may be exacerbated by a combination of changes in the gut microbiota and increased intestinal permeability (16). Evidence for this persistent immune stimulation is supported by both human disease studies and mouse models (17). Cirrhotic livers possess a reduced ability to synthesise innate immune proteins, such as complement and pattern recognition receptors thus reducing its bactericidal capacity. This is further exacerbated by reduced numbers of MAIT cells which have the capacity to respond directly to bacterial metabolites (18, 19).

Compromised immune function not only occurs at a local level in cirrhosis, but also systemically, and affects many different immune cell sub-populations. Neutrophils are reduced, with impaired chemotaxis and subsequent phagocytosis of bacteria as illustrated by a lower response to peptidoglycan recognition proteins (20). Monocytosis is observed in cirrhotic patients with evidence of cellular dysfunction, such as impaired function of the Fc-γ receptors, which are responsible for the clearance of bacteria (21). In alcohol and hepatitis C virus (HCV) related cirrhosis, a reduced frequency of CD27^+^ memory B cell function suggests defective antibody function (22). T cell defects have also been noted including T cell lymphopenia affecting both CD4 and CD8 T cells. There is depletion of naïve and memory T cells, although naïve T cells appear to be more profoundly affected putatively related to splenic pooling, and there is also impaired proliferation of peripheral T lymphocytes (23). NK cells are shown to be lower in number in the periphery but also less responsive to cytokine stimuli which hampers their cytotoxic and anti-fibrotic roles (24). Within the intrahepatic compartment, innate lymphoid cells type 2 (ILC-2) cells appear to be the dominant ILC population (25, 26). The observed changes in immune cell function alters the equilibrium of immunosurveillance and immunotolerance within the liver, promoting the latter through relative immune deficiency. Due to the observed microenvironmental shift towards immunotolerance, there is an increase in host vulnerability to tumorigenesis and the occurrence of HCC.

## Natural Killer Cells

Natural Killer (NK) cells are a key part of the innate immune response against viruses and tumours, and more recently, have been shown to participate in the adaptive immune response through cross-talk with dendritic cells and T cells. They make up between 5-20% of circulating lymphocytes, but a much larger fraction (~50%) of the intrahepatic lymphocyte compartment. NK cells are usually characterised as CD3^-^CD56^+^ lymphocytes and in general do not require priming to initiate anti-viral or anti-tumoral cytotoxic or cytokine secretory effects (27). Recent work has identified that they also have adaptive properties (28), which can be both receptor or cytokine driven. Particular interest has been generated in the utility of cytokine-induced memory NK cells as agents for immunotherapy (29).

Surveillance of diseased cells can be mediated through the ‘missing self’ model (loss of inhibition) or through recognition of stress-induced molecules (gain of activation) (30). The net effects of both of these mechanisms is a change in the balance between activating and inhibitory signals transduced by the NK cell such that activation is favoured. Both these mechanisms may operate in cancer. In the missing-self model, healthy cells which express major histocompatibility complex (MHC) class I are spared from lysis through the engagement of inhibitory receptors on the NK cell surface, such as killer cell immunoglobulin-like receptors (KIR) or CD94:NKG2A. Thus if MHC class I is downregulated, the tonic inhibitory signal to the NK cell is lost and the cell becomes activated (31). Conversely NK cells can be activated by augmenting activating signals. Key activating receptors include the natural cytotoxicity receptors (NKp30, NKp44 and NKp46) and C-type lectin-like receptors, especially NKG2D. In stressed, transformed or infected cells MHC class I is often downregulated and ligands for NKG2D are up-regulated, shifting the NK cell balance towards activation and tumour lysis (31, 32).

The KIR are MHC class I-specific receptors that perform a fundamental role in self-recognition and in functional “licensing” of NK cells. The tuning of the activity of NK cells may be a more dynamic process than previously considered, which is relevant for NK cells within immunosuppressive tumour microenvironments (33, 34), resulting in induced-hyporesponsiveness. The KIR exhibit an extraordinarily high level of diversity at the gene content and allelic levels. In combination with the diversity of MHC class I ligands, the KIR form a complex immunogenetic network, which has been associated with development and outcomes of cancer. The KIR gene family is found on chromosome 19 and comprises 13 functional KIR genes and 2 pseudogenes (35). Two haplotypes KIR-A and KIR-B have been identified with the former having a fixed gene content but substantial allelic diversity, and the latter haplotype displaying variation in gene content and also allelic diversity. There also is substantial diversity in the frequencies of KIR-A and KIR-B haplotypes amongst different human populations (36). This has been proposed to account for some of the diversity noted in anti-cancer responses. For instance, the activating KIR, KIR2DS2, has been associated with protective responses against acute myeloid leukaemia and other solid tumours including HCC (37, 38). Interestingly, KIR2DS2^+^ NK cells appear to express higher amounts of FcγRIII (CD16), a medium-low affinity IgG receptor essential for antibody-dependent cellular cytotoxicity, providing a potential molecular basis for enhanced protection (39). Improved HCC outcomes have been observed in individuals with different KIR : HLA genotypes including HLA-C group 1, KIR2DS5 and the compound genotypes KIR2DL2: HLA-C group 1, KIR3DS1:HLA-Bw4^80T^ and KIR3DS1:HLA-BBw4^80I^ (38, 40).

## Hepatocellular Carcinoma and Natural Killer Cells

In comparison to other cancers, HCC is relatively cold immunologically with only about 25% having an immune reactive phenotype (41, 42). Individuals with HCC have reduced numbers of NK cells within the periphery and these have lower levels of functionality (43). Within the tumour they are present at low frequencies in contrast to myeloid and other lymphoid cells (44). In HCC accumulation of NK cells in intratumoral tissue, as compared to peritumoural tissue, has increased expression of the activation marker CD49a and an increased CD56^bright^:CD56^dim^ ratio, demonstrating localised differences in these two subtly distinct microenvironments (45). Individuals with higher frequencies of NK cells and enhanced cytotoxic and cytokine secretory functions have improved overall survival in HCC following liver resection (46–48). Patient survival also positively correlates with the frequencies of both circulating and intratumoral NK cells in HCC (49), and in two separate studies the response to sorafenib was better if a higher frequency of intratumoral NK cells was present (48, 50). Conversely, overall survival is worse in individuals with fewer intratumoral NK cells and a higher proportion of CD56^bright^ to CD56^dim^ NK cells. The CD56^bright^ sub-population are considered less mature and have lower levels of cytotoxicity than the CD56^dim^ subpopulation, indicating that NK cell functionality is important in determining the outcome of HCC.

NK cells express multiple activating receptors and therefore can engage many different molecules expressed by tumours. Changes in the balance of expression of activating and inhibitory receptors can determine NK cell function in a “rheostat” model. Thus, in advanced HCC there may be upregulation of the inhibitory receptor NKG2A and this, combined with a reduction in the effector molecules perforin and granzyme B, contributes to a hypofunctionality of NK cells (43, 46). Down-regulation of granzyme B is a feature of intra-tumoral, as opposed to peri-tumoral, NK cells and correlates with expression of IL-10-positive tumour-associated macrophages (51). This immunosuppressive gradient also correlates with expression of exhaustion-associated markers such as PD-1, Tim-3, and Lag-3. In addition to modulating T cell functions these molecules may act as checkpoints for NK cells and are often co-expressed on activated or exhausted NK cells (52). CD96 (TACTILE) is another immunological checkpoint associated with HCC, that in combination with its ligand negatively associates with the outcome of HCC (53). Thus, together this group of checkpoints form potential therapeutic targets for HCC.

A number of activating NK cell receptors have been associated with HCC. Differential splicing of NKp30 leads to preferential generation of an inhibitory isoform that predominates in advanced HCC (54). However, most attention has focussed on NKG2D which engages multiple ligands including MIC-A/B and the ULBP proteins. These stress-induced proteins are express on tumours and may be released into the circulation following proteolytic cleavage, through molecules including ADAM9, which is upregulated in HCC (55). Soluble receptors bind and down-regulate NKG2D on the surface of NK cells thus rendering cells less active. In HCC high levels of soluble ULBP1 is associated with poor survival (56). The observation that in mouse models of HCC, NKG2D can drive tumorigenesis, probably through promotion of the chronic inflammation that leads to mutagenesis, indicates the complexity of the molecular pathology of this disease (57, 58). Nevertheless, in general down-regulation of the NKG2D:NKG2D ligand axis is associated with poorer outcomes (59).

## Regulatory T Cells

Sakaguchi and colleagues first described regulatory T cells (Tregs) in the late 1990s (60). Tregs are generated in the thymus. They are a subset of CD4 T cells, expressing high levels of CD25 (the α-chain of the IL-2 receptor) and low levels of IL-7 receptor (CD127). Thus, Tregs are defined by surface receptors as CD4, CD25^high^, CD127^low^ (61). Tregs are crucial in maintaining peripheral immune tolerance (62). Their phenotype and function are controlled by the transcription factor Foxp3 (63). Tregs represent 2–5% of CD4 T cells in humans and about 10% in rodents.

Tregs are present in the human liver. Our group has previously demonstrated that Tregs reside together with effector CD4 and CD8 T cells, CD11c dendritic cells in both interface and lobular hepatitis areas to control liver inflammation (5). The suppressive function of Treg is reduced in the inflamed microenvironment (64). The main function of Tregs is to control autoreactive effector T cells, thereby maintaining hepatic immune tolerance. We have shown that Treg recruitment *via* hepatic sinusoids to inflamed human liver is mediated by the chemokine receptor CXCR3 and the integrin VLA-4 on Tregs and the chemokine ligands CXCL9, 10, 11 and VCAM expression on inflamed sinusoids. Following recruitment, post endothelial migration through the fibrous stromal framework occurs *via* LFA-1 and VLA-4 integrin’s on Treg and cell adhesion molecules ICAM and VCAM on the stroma cells, and subsequently Tregs reside around the area of hepatitis to control liver inflammation (5). Their suppressive function in the human liver is mainly executed *via* CTLA-4, CD39 or IL-10 dependent mechanisms and low dose IL-2 can upregulate functional CTLA-4 on the surface of Tregs surface *via* STAT-5 (65, 66).

Tregs have the potential to be plastic towards effector Th1 or Th17 lineages especially in the inflamed human liver and tumour microenvironment (8). The immune response mediated by T lymphocytes plays an important role in anti-tumour immunity. Tregs secrete immunosuppressive cytokines such as IL-10 and IL-35 to suppress the aberrant immune response, while Th1/Treg and Th17/Treg cells can release not only anti-inflammatory cytokines but also proinflammatory Th1 cytokines such as IFNγ, TNFα, and Th17 cytokines IL-17, IL-22 (67, 68). The ratio of Treg to Th17 cells is closely associated with the outcome of many immune mediated diseases and cancers (69).

## Hepatocellular Carcinoma (HCC) and Tregs

Multiple studies have reported that the frequency of CD4^+^CD25^++^Treg cells in the peripheral blood of HCC patients is significantly higher than in the blood of healthy individuals (70, 71). CD4^+^CD25^++^Tregs in advanced (stage III-IV) HCC patients is significantly increased compared to early (stage I-II) HCC patients, implying that the presence of CD4^+^CD25^++^Tregs is closely related to tumour progression and enhances the invasiveness and metastasis of HCC (71). In addition, accumulation of Tregs correlates with reduced infiltration of CD8 T cells in HCC tumour regions, and the expression of granzymes and perforin functional molecules is less in tumour-infiltrating CD8 T cells (72). Furthermore, in this study an increased quantity of circulating Treg was associated with a high mortality and reduced survival time of HCC patients.

CD4^+^CD25^++^Tregs suppress the anti-tumour immune response either in the draining lymph nodes or in tumour tissue. CD4^+^CD25^++^Tregs in the tumour-draining regional lymph node inhibit the proliferation of effector T cells, and Tregs prevent effector T cells from killing tumour cells in the primary tumour tissue (73). It has been shown that the level of Treg cells in cancer tissue was significantly higher than that in adjacent tissues (74). It is thought that Treg cells may decrease the proliferation of effector CD4 and CD8 T lymphocytes in the tumour microenvironment by contact inhibition, subsequently reducing the anti-tumour immune response and resulting in the potential for tumour cells to escape immune surveillance. HCC is one of the most common and aggressive human malignancies and CD4^+^CD25^++^Treg promote hepatocellular carcinoma invasion *via* TGF-β1 dependent mechanisms (75). Furthermore, ICOS^+^ FOXP3^+^ Tregs contribute to the immunosuppressive HCC microenvironment and lead to an unfavourable prognosis for HCC patients (76). Thus, removal or reduction of the Treg cell population, or inhibition of CD4^+^CD25^++^Treg function in the HCC microenvironment may facilitate the efficacy of tumour immunotherapy (77). In addition, the Th17/Treg ratio is a risk factor for HCC (78). The majority of expanded Tregs do not express CD45RA suggesting that Tregs have a memory phenotype and exponential expansion of these tumour antigen exposed memory Tregs has been a distinct finding in HCC (79).

## Treg and NK Cell Interactions in Hepatocellular Carcinoma

**Figure 1:**
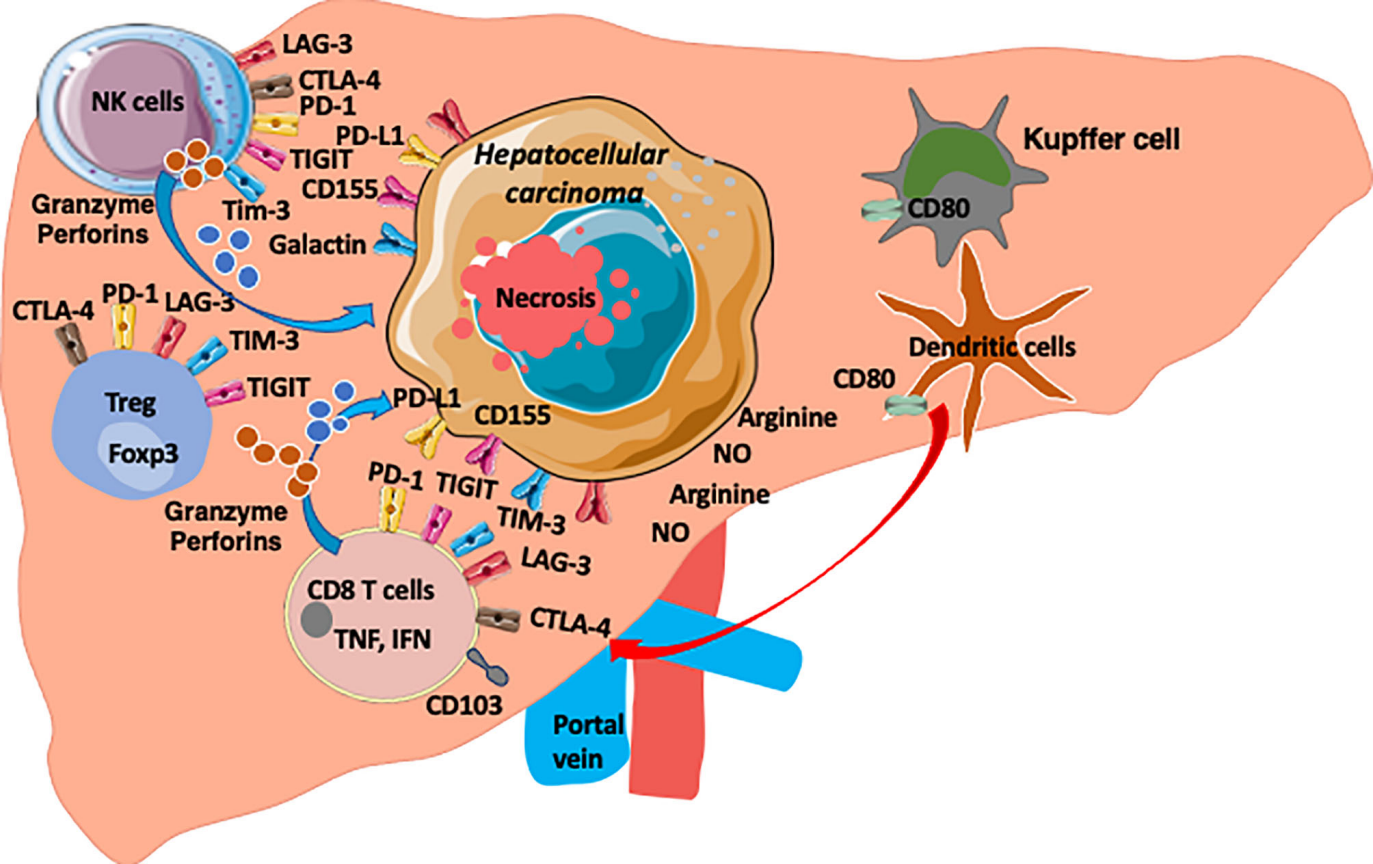
The HCC microenvironment is enriched with anti-tumour CD8 T cells, NK cells and Treg cells. Both T cells and NK cells express checkpoint molecules (PD1, TIGIT, TIM3, LAG3 and CTLA-4) as common targets. HCC express ligands for these checkpoints (PDL1, CD155, Galactin). Arginine and Nitrous oxide are also enriched in HCC environment and the microenvironment is hypoxic environment and enriched with chemokines, cytokines, metabolites and microbial products (8).

The balance of NK cells which provide tumour clearance and Tregs which inhibit tumour immunity may determine the outcome of HCC (**Figure 1**). A potential role for Tregs in dampening NK cell functions was first suggested in a murine leukaemia model. In this model, the depletion of Tregs by administration of anti‐CD25 mAb before tumour inoculation abolished tumour growth and promoted the generation of cytotoxic cells characterized as NK cells (80). In HCC, the NK cell frequency within the tumour-infiltrating lymphocyte compartment is less than in the non-tumour tissue, and these intratumoral cells demonstrate impaired cytotoxicity and IFN-γ production (43, 81), We have also previously demonstrated that there is a parallel increase in NK cells and Treg in hepatic inflammation (82) suggesting that taming the Treg population will allow NK cells to function more efficiently.

Freshly isolated human Tregs can directly inhibit human NK cell cytotoxicity against K562 (83). TGF-β maintains the inhibitory functions of Treg and plays a suppressive role by inhibiting the expansion of NK cells and their cytotoxic functions (84, 85). TGF-β can also have a negative effect by facilitating the onset of tumours due to a reduction of immunosurveillance and anti-cancer responses. Resting NK cells harbour surface expression of TGF‐β receptors, rendering them susceptible to soluble TGF‐β (86). Resting human Tregs express membrane‐bound TGF‐β that is associated with the protein, latency‐associated protein (LAP) (87). When associated with LAP, TGF‐β remains inactive. Membrane‐bound TGF‐β is involved in the inhibitory function of Tregs on NK cells since anti‐TGF‐β blocking antibodies can restore the cytotoxicity IL‐12‐induced IFN‐γ secretion of human NK cells (88). When exposed to TGF‐β or on Treg encounter, Smad signalling in NK cells blunts expression of cytotoxic molecules such as granzyme B and perforin (89). In addition, high levels of TGF-β have been associated with impaired NK cell function and NKG2D expression (90). Therefore, Tregs bearing TGF-β on their membrane can present it directly to NK cells resulting in a reduction in NKG2D expression (88). Activated Tregs can also suppress NK cells responses *via* an IL-2 mediated mechanism that is crucial for NK cell survival (91). This reduces the capacity of NK cells to secrete IFN‐γ on stimulation with IL‐12 but not on activation by IL‐2 and IL‐15 suggesting the regulation of NK cell control by Tregs is critically dependent on the cytokine milieu in the HCC microenvironment.

## The Influence of the Tumour Microenvironment on NK Cell Function

The intrahepatic microenvironment is crucial for both NK and Treg phenotypic stability, functionality, survival and proliferation (8). The HCC microenvironment is an active component of the tumour rather than merely a passive structural support for tumour growth, which changes dynamically and consequently affects HCC behaviour. These immunosuppressive features of HCC are a challenging barrier to clinicians to design effective immunotherapies. The HCC microenvironment is composed of not only growth factors, cytokines, metabolites and chemokines generated by stroma and tumour cells, but also tumour-infiltrating macrophages, myeloid-derived suppressor cells, neutrophils, cancer-associated fibroblasts and regulatory T cells. They all play key roles in the clinical outcome of HCC and success or failure of immunotherapies.

Hepatic stellate cells (HSCs) are the major framework of HCC and can directly promote tumour cell proliferation. Conditioned medium collected from HSCs induce not only proliferation and migration of HCC cells but also promote HCC growth through the activation of NF kappa B and extracellular-regulated kinase (ERK) pathways (92). Cancer-associated fibroblasts are the major cell type within the tumour stroma and play a critical role in tumour-stromal interactions (93). They are activated by TGF-β and are responsible for the synthesis, deposition and remodelling of excessive extracellular matrix thus modulating the biological activities of HCC. HCC cell growth, extravasation and metastatic spread are dependent upon the presence of these fibroblasts. HCC cells can reciprocally stimulate proliferation of tumour associated fibroblasts, suggesting their key role in tumour-stromal interaction (94). Stroma from HCC express several growth factors, including hepatocyte growth factor (HGF), epidermal growth factor (EGF), fibroblast growth factor (FGF) and Wnt family members, stromal-derived factor (SDF)-1α and IL-6 (95).

Additionally, myeloid derived suppressor cells (MDSC) exert multiple mechanisms of immunosuppressive activity in the tumour microenvironment. MDSCs induce differentiation and expansion of Tregs during tumorigenesis; inhibit DCs and NK cells *via* TGF-β; deprive T cells of essential amino acids such as L-arginine and L-cysteine; and generate the oxidative stress that is associated with HCC progression (96). MDSCs co-cultured with autologous T cells induce an increased number of Tregs, PD-1^+^-exhausted T cells, and an increase in immunosuppressive cytokine levels in HCC patients (97). MDSCs can also impair NK cell function. In HCC, MDSCs inhibit NK cell cytotoxicity and cytokine release mediated by the NKp30 receptor (98). MDSCs also inhibit TLR-ligand-induced IL-12 production and inhibit the T-cell stimulating activity of DCs in HCC (99). Tumour-associated neutrophils can also recruit macrophages and Treg cells into HCCs to promote their growth, progression, and resistance to sorafenib therapy (100, 101).

Furthermore, immunosuppressive cytokines, extracellular matrix and inflammatory cytokines in the HCC microenvironment can define HCC biology and prognosis. Global gene expression profiling of human HCC indicates that TGF-β gene signatures cluster HCC into two homogeneous groups with early or late TGF-β signatures (102). Importantly the late TGF-β signature is associated with an invasive HCC phenotype and an increased risk of tumour recurrence. MMP1 and TIMP1 were also signature genes in the immature hepatoblast subtypes of HCC that is associated with a poor prognosis (103). Inflammation-associated pathways, gene expression signatures, NF-κB, TNF-α, and IL-6 from the adjacent benign tissue can also predict late recurrence of HCC (104). IL-6, a major pro-inflammatory cytokine, is one of the signature genes in the hepatoblast phenotype signature (103). Osteopontin, secreted from Kupffer or stellate cells in response to inflammatory cytokines, is also associated with metastasis of HCC (105).

The tumour microenvironment also shapes NK cell metabolism and effector functions. Understanding immunometabolic suppression is critical in engineering a new generation of effective natural killer cell-based immunotherapies targeting solid tumours such as HCC. Multiple factors can modulate NK cell metabolism in the tumour microenvironment. HCC exert immunosuppressive effects through a number of mechanisms, a key driver of which is hypoxia. High oxygen consumption by tumour cells can generate hypoxic regions. Hypoxia impairs NK cell effector functions, but also sustains HIF1α, which promotes glycolytic metabolism. Hypoxia fuels the generation of adenosine from the cancer-associated ectoenzymes CD39, expressed on Treg (106), and CD73 on antigen presenting cells. Tumour cells also generate extracellular adenosine through the CD39 and CD73 ectonucleotidases, thus compromising NK cell function through competition for nutrients.

Thus, the interaction between the tumour and stroma interaction generates the microenvironment within HCC tissue and can suppress the effect of surrounding tissues or cell types that stimulate hepatocarcinogenesis, tumour progression, invasion, and metastasis. Sorafenib, an oral multi-kinase inhibitor, which is the most widely used HCC medication, inhibits VEGFR-2/-3 and PDGFR as well as Raf kinase, disrupting tumour-stromal interactions and resulting in decreased cell proliferation and angiogenesis. The efficacy and safety of sorafenib have been demonstrated in Phase III clinical trials, and it is currently the standard of care for patients with advanced stage HCC (104, 107).

## Immunotherapeutic Interventions and How they May Enhance NK Function and Tame Treg Suppression

Currently the treatment for HCC is challenging, often due to the stage at which many patients present. Therapeutic options include surgery, transplantation and locoregional therapies can be curative, and systemic therapies with tyrosine kinase inhibitors including sorafenib and lenvantinib result in a modest prolongation of survival. As a result there is much interest in novel therapeutics and therapeutic combinations which have been recently reviewed by Llovet et al. (108).

In terms of immunotherapy, trials of checkpoint inhibitors have been the best studied. The PD-1 checkpoint inhibitors nivolumab and pembrolizumab have been used for the treatment of patients with HCC (109), but only leads to clinical responses of 10-20%. Several factors including the expression of programmed cell death-Ligand 1 (PD-L1), tumour mutational loads, and tumour-infiltration immune cells correlate with patient responses using these medications (110). This relative lack of efficacy implies that combination therapy should be considered and applied to each patient as personalized treatment approaches in HCC. Consistent with this combining a PDL1 inhibitor (Atezolizumab) with an anti-VEGF antibody (Bevacizumab) increased survival for unresectable HCC by approximately six months compared to sorafenib monotherapy (111). As checkpoint inhibitors predominantly target T cells, one attractive approach is to simultaneously target NK cells thus effectively mobilizing two arms of the immune system.

### Enhancing NK Cell Frequency and Function

The field of NK cell therapeutics is rapidly growing. The observations of Ruggeri et al. demonstrating that NK cell alloreactivity was beneficial in refractory leukaemia acted as the cornerstone for the development of NK cell-based approaches (112). Initial work focussed on culturing NK cells *in vitro* and then infusing them, met with success mainly for haematological malignancies rather than solid tumours (113). However, this may be augmented by combining NK cells with monoclonal antibody therapy thus targeting the ADCC function of NK cells, or by genetically modifying NK cells to express chimeric antigen receptors (CAR-NK cells). Both these strategies have reached clinical trials (114, 115). Encouragingly the use of CD19-transduced CAR-NK cells did not result in the increase in cytokine levels associated with the systemic toxicity of CAR-T cell therapies. An alternative strategy is to target inhibitory receptors. As NK cells are held in check by dominant inhibitory signals then they are excellent candidates for having their activity unleashed by blocking these inhibitory receptors using monoclonal antibody therapeutics. These have targeted both the KIR receptors and NKG2A. Importantly, the clinical effects of the anti-NKG2A monoclonal antibody Monalizumab may be related to unleashing both T and NK cells for its anti-tumour effects (116).

In current clinical trials of adoptive tumour immunotherapy, large dosages of NK cells have been used ranging between 5 x 10^6^ to 5 x 10^7^/kg body weight (117). An approach to achieve these large numbers of NK cells is *via* the enrichment of NK cells from donor-derived leukapheresis products. Multiple protocols have been successfully developed to generate GMP NK cell products through immunomagnetic depletion of T and B cells and positive selection of CD56^+^ cells (118). The necessity of the NK cell products to be of a high-purity, which requires not only a long manufacturing process and compromises the viability and potency of the NK cell product, combined with the limited availability of autologous leukapheresis products makes the task of obtaining sufficient GMP NK cells from a single leukapheresis challenging. Therefore, an alternative method is to make NK cell products *via* the expansion of NK cells from PBMCs using feeder cells. An example of this are K562 cells modified with membrane-bound molecules such as IL-15 and 4-1BB ligand which can rapidly expand NK cells from PBMCs by 21.6-fold in 7 days (119). This method also produces NK cell products with purities in the range of 60-70%. To achieve purities needed for allogenic use a further expansion up to 21 days or enrichment of NK cells is still required (117, 119). NK cells can also be derived from induced pluripotent stem cells (IPSCs) and this has the advantage of hugely increasing their availability, as well as the prospect of selecting NK cell populations based on their alloreactivity potential, which may enhance their anti-cancer response (120).

IL-2 can also be used for short term activation of NK cells without feeder cells. IL-2 activation of NK cells can be coupled with CD3^+^ T-cell and CD19^+^ B cells depletion to increase the purity of the cell product (121). GMP NK cells have high IFNγ expression upon cultivation with K562 tumour cells and are highly cytotoxicity toward tumour cell lines *in vitro* (122). These data confirm that NK cells can have high clinical potency and potential for a significant role in tumour immunotherapy, including HCC.

**Figure 2:**
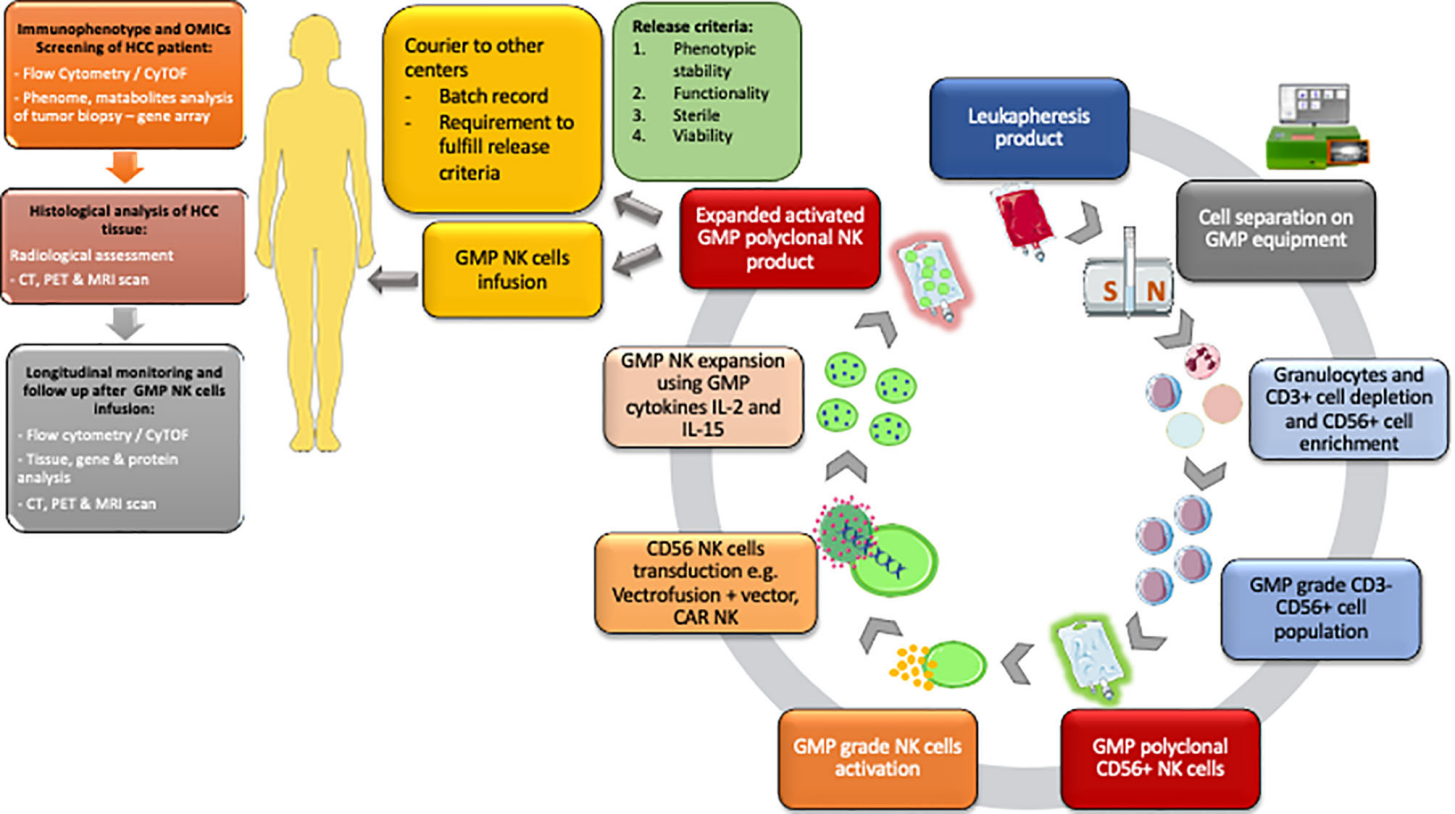
Workflow for the isolation of GMP polyclonal NK product and subsequent pathways from the NK cell isolation to distribution to other centres. GMP NK cell product must fulfil the release criteria according to MHRA criteria. Flow cytometry/CyTOF and histological analysis, CT, PET and MRI scans are performed before cell infusion to help identify individuals for therapy. Following GMP NK cell infusion the patient immune system is monitored using a combination of flow cytometry/CyTOF, tissue analysis and cross-sectional radiological imaging. HCC, Hepatocellular carcinoma; OMICs, genomic proteomic metabolomic; CyTOF, cytometry time of flight.

Many companies have developed GMP cell isolation equipment and GMP reagents and have a manufacturing platform which has been utilised for multiple clinical trials utilising NK cell therapy (**Figure 2**). There are currently only a few MHRA and HTA approved GMP facilities in the UK focusing on GMP T cells and NK cell therapy for patients with both autoimmune diseases and cancer. GMP grade magnetic isolation or GMP cell sorting of clinical grade CD56-positive NK cells and applying these cells as immunotherapy in HCC could be one of the future treatment for these patients.

### Suppressing Treg Frequency and Function in HCC

Restraining Tregs in HCC is important for many reasons. Anti-tumour T cell responses are severely compromised in advanced HCC patients through multiple immunosuppressive pathways comprising Tregs, PD-1^+^ T effector cells, and inhibitory cytokines (72, 97, 123). In HCC, expression of PD-1 is increased on CD8 memory T effector cells and interaction with its ligand PD-L1 on HCC cells blocks signalling, proliferation, and cytokine secretion of anti-tumour CD8 T cells (124, 125). The association between the infiltration of CD8 T cells in HCC and patient survival has been well recognised (126, 127). Granzyme B, perforin and IFNγ secretion by CD8 T cells generates potent anti-tumour activity but high expression of PD-1 on exhausted T cells contributes to ineffective effector T cell function, and selective *in vitro* depletion of these immunosuppressive cells results in improvement of T effector cell function in HCC patients (128, 129).

Activated Tregs also have the ability to inhibit effector T cells *via* contact-dependent interactions between checkpoint molecules and their ligands including PD-1 with PD-L1, Tim-3 with galectin-9, CTLA-4 and GITR. Tregs also contribute to the strongly immunosuppressive HCC microenvironment by releasing the inhibitory cytokines TGF-β and IL-10 (130, 131). The mechanism of inhibition of anti-tumour effector T cells by Treg involves several molecular pathways: 1) Tregs may inhibit proliferation and cytokine secretion of T effector cells by IL-10, adenosine production from CD39 on its surface and IL-35, which can be reversed by adding neutralising antibodies (132, 133); 2) *via* the PD-1/PD-L1 pathway to suppress anti-tumoral immunity in HCC (134); and also *via* 3) the co-inhibitory molecule CTLA-4 (135).

### Suppressing Tregs and Boosting NK Cells in HCC

**Figure 3:**
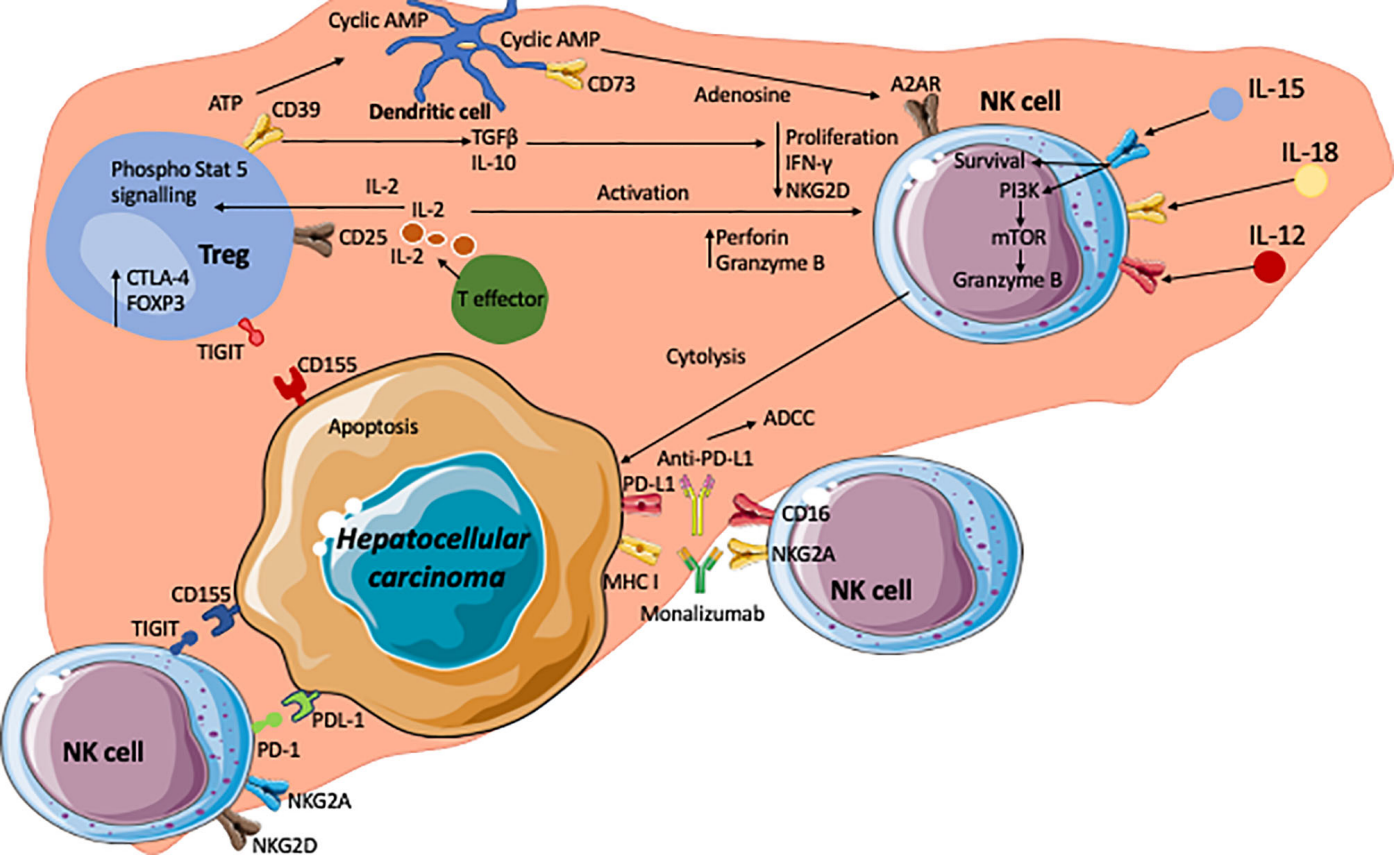
The hepatocellular carcinoma microenvironment and crosstalk of NK, Treg and HCC with the key signalling cascades. Effector T cells are the source of interleukin-2 (IL-2) which acts on the IL-2α receptor in CD25 on regulatory T cells. This leads to phosphorylation of STAT5 which subsequently upregulates Treg functional molecule CTLA-4 and the transcription factor Foxp3. The tumour microenvironment is enriched with adenosine triphosphate (ATP). CD39 on Tregs generates cyclic AMP from ATP. CD73 expressed on intrahepatic Tregs subsequently generate immunosuppressive adenosine from the cyclic AMP. Adenosine act on A2AR on NK cells which leads to the suppression of NK cell function. In addition, immunosuppressive cytokines TGFβ and IL-10 are secreted by Tregs and these cytokines lead to reductions in proliferation, IFNγ production and expression of NKG2D. Conversely, high concentrations of IL-2 lead to an increase in perforin and granzyme B expression on NK cells. IL-12, IL-15 and IL-18 act on their corresponding receptors on NK cells and leading to enhanced cell survival, and granzyme B production *via* PI3Kinase and mTOR pathways. NK cells express inhibitory receptors such as TIGIT and PD-1, which interact with corresponding ligands CD155 (PVR) and PDL-1 expressed on tumour cells. Inhibition of these receptors with check point inhibitors could lead to unleashing of NK cell cytotoxic activity to tumour cells *via* secretion of granzymes, perforin, IFNγ and TNFα. Monoclonal antibodies against inhibitory receptors such as immune checkpoint inhibitor monalizumab are developed to block MHC-I ligands and NKG2A interactions and enhance NK cell cytotoxicity towards cancer cells. CD16 receptors on NK cells allows them to carry out ADCC targeting such molecules as PD-L1.

Combination of these approaches by inhibiting Tregs and enhancing NK cells is an exciting option which has not been tried before (**Figure 3**). One of the potential approaches would be sequential manipulation by administering Basilizumab (anti-CD25) to deplete CD4^pos^ CD25^high^ Treg cells followed by GMP NK cells either *via* a peripheral route or direct administration along with transarterial chemoembolization (TACE) as GMP-NK TACE therapy. This would prevent systemic depletion of Treg thus reducing the potential for inducing autoimmunity. With the remarkable success of chimeric antigen receptor (CAR)- engineered technology, developing CAR-engineered NK (CAR-NK) cells for cancer therapy could offer some significant advantages, including better safety by a lack or minimal cytokine release syndrome, multiple different mechanisms for inducing cytotoxic activity including checkpoint inhibition, and off-the-shelf manufacturing. CAR-NK cells could also have better infiltration into solid tumour such as HCC and overcome the resistant tumour microenvironment.

Targeting intratumoral Treg cells may offer a therapeutic direction to modulate the tumour microenvironment. The combination of nivolumab with the Treg-depleting anti-CCR4 antibody, mogamulizumab has been explored by Doi et al. (136). In their proof of concept study this combination provided anti-tumour activity and can thus be a potentially effective option in cancer immunotherapy. A recent study found that CD36 was selectively upregulated in intratumoral Treg cells as a central metabolic modulator. They genetically ablated *CD36* in Treg cells resulting in suppressed tumour growth, a decrease in intratumoral Treg cells and enhanced antitumour activity (137). In addition, a recent report identified widespread *HLA-E* expression in tumour samples, with levels correlating to those of *NKG2A*. This is of importance as one mechanism of tumour resistance to immune cells is mediated by the expression of peptide-loaded HLA class I molecule (HLA-E) in tumour cells. HLA-E suppresses NK cell activity *via* ligation of inhibitory receptor, NKG2A (138). Furthermore, blockade of NKG2A results in the enhancement of tumour immunity by promoting both NK and CD8+ T cell effector functions in mice and humans. As described above, monalizumab is a humanised anti-NKG2A antibody which has been shown to enhance the activity of NK cells against various tumour cells and rescue CD8+ T cell function in combination with blockade of the PD-x axis (116).

## The Potential for Future Combination Immunotherapy in HCC

### Checkpoint Therapy to Enhance T and NK Cell Function

Both T cells and NK cells express co-inhibitory molecules or checkpoint inhibitors, which can be targeted using CPI to unleash potent anti-tumour immunity by recovering both NK and T cell function (116, 139). These include PD-1, CTLA-4, TIGIT and LAG-3. The mechanism of action of PD-1 involves the engagement of its ligands PD-L1 and PD-L2 to deliver inhibitory signals that regulate the balance between T cell exhaustion, tolerance and immunopathology (140). Tumour cells expressing PD-1 ligands on their surface use the PD-1 pathway to attenuate tumour immunity and facilitate tumour progression (141). PD-1/PD-L1 has been recognised to play a role of critical importance in immune escape in HCC after the successful treatment of nivolumab in patients with advanced HCC, achieving an objective response rate of 15-20% (142). Furthermore, elevated PD-L1 expression in HCC significantly correlates to poor survival and tumour aggressiveness (143–146). Blockade of the PD-1 and PD-L1 interaction using monoclonal antibodies produces durable clinical responses in patients with diverse advanced tumour types (147).

CTLA-4 outcompetes the co-stimulatory molecule CD28 for binding to B7-1/CD80 or B7-2/CD86 expressed on the surface of antigen presenting cells, including tumour infiltrating dendritic cells. This is due to its higher affinity for CD80/CD86 as compared to CD28. As a result of this, CTLA-4 negatively regulates T-cell activation, inactivating T lymphocytes in the G1 phase. CD8 T lymphocytes are able to exert their anti-tumour cytotoxic effects *via* the secretion of TNFα and IFNγ leading to the apoptosis of tumour cells (148), when the T-cell receptor (TCR) binds its cognate peptide:MHC antigen expressed on tumour cells (149–153). CTLA-4 blockade can then provide long-lasting tumour remission due to its impact on memory T cells response to cancer (154). After blocking CTLA-4, an increase in the number and breadth of protective T cells can be seen in the blood as evidenced by TCR V-β analysis (154). However, its role in the anti-tumour NK cell response requires further study.

TIGIT (T cell immunoglobulin and ITM domain) is an inhibitory receptor and is expressed on activated T cells and can also be found on NK cells as well as memory T cells, a subset of Treg cells as well as follicular T helper (Tfh) cells (155–160). TIGIT is recognised for its protective role in autoimmune diseases as well as cancer. To date, tumour associated lymphocytes expressing TIGIT have been shown to exist in acute myeloid leukaemia, non-small cell lung cancer, colo-rectal carcinoma and melanoma (161–163). TIGIT is a key checkpoint inhibitor in anti-tumour responses and thus presents a promising target for future immunotherapies (161). TIGIT binds to its ligand PVR or CD155 on the tumour cells with a much higher affinity than its activating counterpart CD226 (DNAM-1), thereby inhibiting the interaction between CD226 and CD155 which is widely expressed on tumour cells (158–160, 164). CD226 promotes cytotoxicity and enhances anti-tumour responses (165, 166), whereas TIGIT, which outcompetes CD226, negatively regulates anti-tumour responses (167). After TIGIT/CD155 ligation, TIGIT’s immunoglobulin tail tyrosine-like motif becomes phosphorylated at Tyr225 and binds to cytosolic adapter Grb2. This in turn leads to NK cell immunosuppression and dysfunction, including downregulation of IFN-γ production (168). It is now recognised that TIGIT expression on tumour-infiltrating NK cells is associated with tumour progression and was also linked to functional immune exhaustion (139). The role of TIGIT in liver cancer is still under investigation. Recent research has shown that survival and prognosis of HCC patients is positively correlated with NK cell numbers in blood and tumour tissue (169). Tumour progression of these HCC patients was associated with dysfunction of the tumour-infiltrating NK cells (170), and exhausted tumour-infiltrating NK cells correlate with poor clinical outcome for HCC patients. Importantly, this NK cell exhaustion was reversed by manipulating the TIGIT pathway (171). Thus, the clinical application of anti-TIGIT CPI immunotherapy will enhance the NK cell anti-tumour immune response and is a promising new approach towards treating HCC.

Lymphocyte activation gene-3 (LAG-3) belongs to the immunoglobulin superfamily and is expressed on tumour infiltrating lymphocytes (TILs) (172), NK cells (173), B cells (174) and DCs (175). LAG-3 has a high binding capacity to MHC II (173). Current data suggests that modulating LAG-3 can impact autoimmunity, cancer and chronic viral infection (164). Fibrinogen-like protein 1 (FGL1) is a new major ligand for LAG-3 and it has recently been demonstrated that blocking the FGL1-LAG-3 pathways results in the stimulation of tumour immunity and inhibits tumour growth (176). Furthermore, co-expression of LAG-3 and PD-1 on TILs has been observed in several mouse tumour models. One of the first pre-clinical cancer models using anti-LAG-3 demonstrated enhanced activation of tumour-specific T cells at the tumour site and disruption of tumour growth, especially when used in combination with anti-PD-1 (177). Currently human studies involving LAG-3 as a target are usually performed in combination with PD-x axis blockade, providing a wider checkpoint blockade than monotherapy.

The use of monoclonal antibodies targeting CTLA-4, PD-1 and PD-L1 has seen excellent success, especially in settings such as melanoma and non-small-cell lung cancer. However, most HCC patients have underlying cirrhosis, and there is a concern about the risk of decompensation related to CPI-induced immune mediated hepatitis. This appears relatively rare occurrence during checkpoint inhibitor therapy for HCC with grade ≥3 side effects affected under 5% of patients (142, 178, 179). Nivolumab and pembrolizumab have now received approval from the FDA as second-line treatments for advanced HCC based on both the Checkmate 040 (142) and Keynote 224 (178) clinical trials. Although subsequent phase III trials have failed to show statistically significant data for survival improvement in either first-line (nivolumab vs. sorafenib) or second-line (pembrolizumab vs. placebo) setting (179, 180). These therapeutics now have potential to be combined with anti-VEGF therapies (111), however there remains an unmet clinical need for investigating combinatorial blockade targeting the novel inhibitory receptors, such as TIGIT and LAG-3.

### Combination of GMP NK Infusion and Check Point Inhibitors in HCC

NK-cell therapy in cancer has made significant progress in the past decade with many milestones. It has been shown that NK-cell alloreactivity can eliminate the risk of leukaemia relapse and graft rejection as well as protecting against graft-versus-host disease in transplant patients (112). High-risk myelodsysplastic syndrome (MDS) patients have been shown to be responsive to NK-cell therapy due to the infused donor NK cells causing a reduction in high-risk clones and a less pronounced host immune activation (181). The infusion of NK cells has also been successful in patients with refractory acute myeloid leukaemia achieving remission in one third of patients (113). A recent phase I trial investigating the use of NK-cell therapy in combination with trastuzumab in HER2-positive cancer patients demonstrated that the therapy was well tolerated and that target engagement and anti-tumour activity was seen in the patients (115). CAR-NK cell infusion is also an exciting and promising therapy for cancer patients with a recent phase I and II trial showing that the majority (8 out of 11) patients responded to the treatment, of which 7 patients had complete disease remission (114). Recent developments suggested that NK cells derived from induced pluripotent stem cells (iPSCs) produce inflammatory cytokines and exert strong cytotoxicity against a variety of hematologic and solid tumours. iPSC-derived NK cells were also found to recruit T cells and cooperate with T cells and anti-PD-1 antibody, subsequently enhancing inflammatory cytokine production and promoting tumour lysis (120). Additionally, NK-CAR-iPSC-NK cells have been shown to significantly inhibit tumour growth resulting in prolonged survival in an ovarian cancer xenograft model (182).

Anti-PD-1, anti-PD-L1 and anti-CTLA-4 monoclonal antibodies also enhance NK cell tumour trafficking and release cytokines against tumours whilst simultaneously supressing Treg function (183–186). Anti-CTLA-4 monoclonal antibodies have also been shown to induce the release of TNFα against tumour cells *via* CD16 binding to antibody-bound tumour cells, and simultaneously to induce Treg inactivation (187–189). Blocking TIGIT and its ability to exploit both T cell and NK cell responses is also a strategy that is currently being explored by multiple pharmaceutical companies undergoing phase I/II clinical trials. Experimental drugs such as tiragolumab (anti-TIGIT) is currently undergoing phase I trials in various cancers (https://clinicaltrials.gov/ct2/show/NCT02913313) in combination with atezolizumab and nivolumab. Specific clinical trials looking at patients with HCC include looking at the use of SRF388 which is a fully human IgG1 antibody against IL-27 that decreases the expression of inhibitory immune checkpoint receptors (https://clinicaltrials.gov/ct2/show/NCT04374877?term=TIGIT+HCC&draw=2&rank=).

Thus, combining the massive increase in CPI therapy with the growth in adoptive NK cell therapeutics indicates that there is now a great potential to generate novel therapeutic combinations that target both CD8 T cells, NK cells and Tregs generating a holistic approach to cancer immunotherapy. Such an approach could simultaneously positively modulate the tumour microenvironment and directly cytotoxicity against tumours. In particular, the combination of augmenting both CD8 T cells and NK cells means that tumours that down-regulate MHC class I to avoid CD8 T cell mediated lysis, lose an important inhibitory signal for NK cells, and thus render themselves susceptible to NK cell killing. However, new therapeutic combinations have the potential to exacerbate toxicity, especially those related to autoimmunity and so these approaches need caution. In general, though NK cell therapeutics have proven relatively safe with little systemic toxicity observed, so these combinations may be more advantageous than those that target solely T cells. Nevertheless as with all therapeutic combinations it is important that future studies are carefully monitored for signs of unexpected toxicity.

## Conclusion

HCC remains a challenge for clinicians and researchers to approach in partnership. The recent development of multiple new immunotherapies including monoclonal antibodies and cell products present new opportunities for the clinician to treat HCC. However, understanding the immunological microenvironment in which HCC occurs will be key to successfully combining these. Fundamental research is required to unpick the different intrahepatic immunological microenvironments on which HCC occurs. Understanding these on a personalized basis will be the key to selecting the optimal combination of immunotherapies for each patient.

## Author Contributions

AB and FW contributed equally to this work. All authors contributed to the article and approved the submitted version.

## Funding

We received funding from Sir Jules Thorn Trust Biomedical Research Grant, Transbioline Innovative Medicine Initiative Research Grant, Queen Elizabeth Hospital Birmingham Charity, Medical Research Foundation - G1002552 from the The Medical Research Council (grants M019829 and S009338), CRUK (“HUNTER” accelerator award) the NIHR Birmingham BRC, and the NIHR Southampton BRC.

## Conflict of Interest

The authors declare that the research was conducted in the absence of any commercial or financial relationships that could be construed as a potential conflict of interest.
